# Pulmonary artery mass with *PIK3CA* mutation after orthotopic heart transplantation

**DOI:** 10.1186/s13019-024-03090-2

**Published:** 2024-10-01

**Authors:** Andre Y. Son, Aaron J. Clark, Borislav A. Alexiev, Duc Thinh Pham

**Affiliations:** 1 Division of Cardiac Surgery, Northwestern University Feinberg School of Medicine and Northwestern Medicine, 676 N St Clair Street Suite 730, Chicago, IL 60611-2968 USA; 2 Department of Pathology, Feinberg School of Medicine, Northwestern University, Evanston, USA

**Keywords:** Pulmonary artery mass, Heart transplantation, Minimally invasive surgery

## Abstract

**Background:**

Patients can develop de novo malignancies following orthotopic heart transplantation. However, vascular tumors are not commonly described in this population.

**Case presentation:**

We present a 69-year-old female with a history of orthotopic heart transplantation for chemotherapy-induced cardiomyopathy who developed an incidental pulmonary artery mass six years after her transplantation. Given concerns for malignancy, the patient underwent an operative excisional biopsy through a left anterior mini-thoracotomy with femoral artery and vein cannulation for cardiopulmonary bypass. The mass was determined to be a non-malignant vascular overgrowth with *PIK3CA* mutation.

**Conclusion:**

We present the case of an unusual pulmonary artery mass with *PIK3CA* mutation found in a post heart transplant patient. We were able to spare her the morbidity of a redo-sternotomy by excising the mass via a minimally invasive left anterior thoracotomy approach.

## Introduction

Heart transplant recipients are at increased risk of developing de novo malignancies following transplantation, with up to 10% developing malignancy 1–5 years after transplantation [1]. Moreover, patients with pre-transplantation malignancies have higher risk for developing posttransplant malignancies [2]. Therefore, post-transplant patients with findings concerning for malignancy should undergo diligent investigation. We present the case of a post-heart transplant patient who developed a metabolically active pulmonary artery mass concerning for sarcoma that was found to be a non-malignant vascular overgrowth with *PIK3CA* mutation.

## Case report

**Fig. 1:**
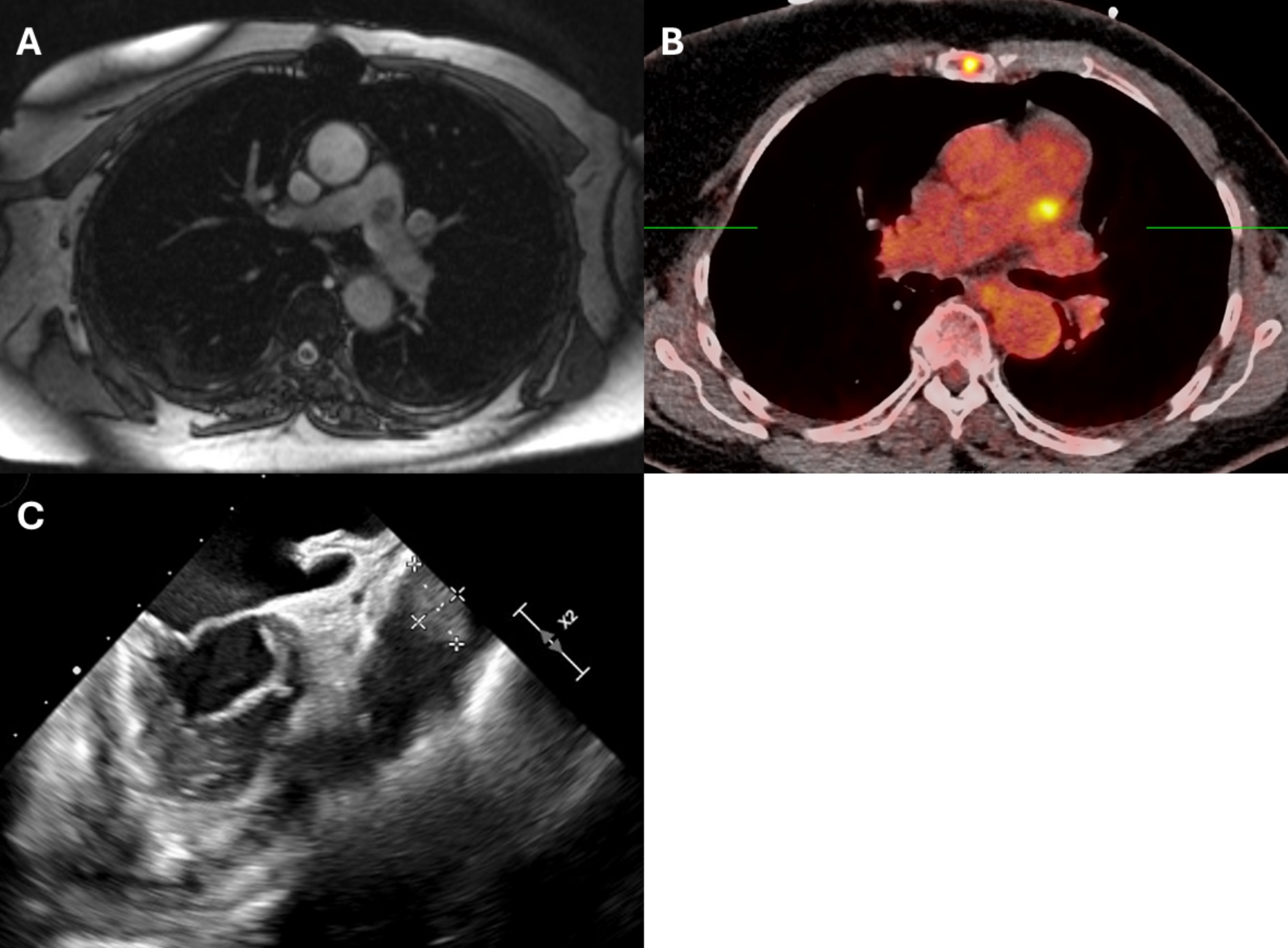
Diagnostic imaging of pulmonary artery mass shown by (**A**) cardiac magnetic resonance imaging; (**B**) PET computed tomography; (**C**) transesophageal echocardiography

A 69-year-old female with a history of grade 3, triple positive, multifocal invasive ductal carcinoma of the left breast cancer 17 years ago, underwent left mastectomy and adjuvant treatment with Adriamycin, cyclophosphamide, paclitaxel, and Herceptin. Unfortunately, her course was interrupted after a year when she developed Adriamycin and Herceptin-induced cardiomyopathy and heart failure. She had an automatic implantable cardioverter defibrillator placed 14 years ago, then underwent a heart transplantation 6 years ago. She was in good health with good graft function on echocardiograms and no evidence of rejection on post-transplant biopsies. Her immunosuppression regimen consisted of tacrolimus and mycophenolate mofetil. Six years after her transplant, she underwent a surveillance cardiac MRI with stress to evaluate coronary perfusion. The study showed normal myocardial perfusion without inducible ischemia; however, there was an incidental finding of a 1.8 × 1.7 cm lesion in the main pulmonary artery (Fig. 1A). A subsequent PET CT was performed, showing metabolic activity with SUV max of 5.2 and adherence to the main pulmonary artery, suggestive of a malignancy, possibly sarcoma (Fig. 1B). Given these findings, the decision was made within several months to undergo open heart surgery to perform an excisional biopsy.

**Fig. 2:**
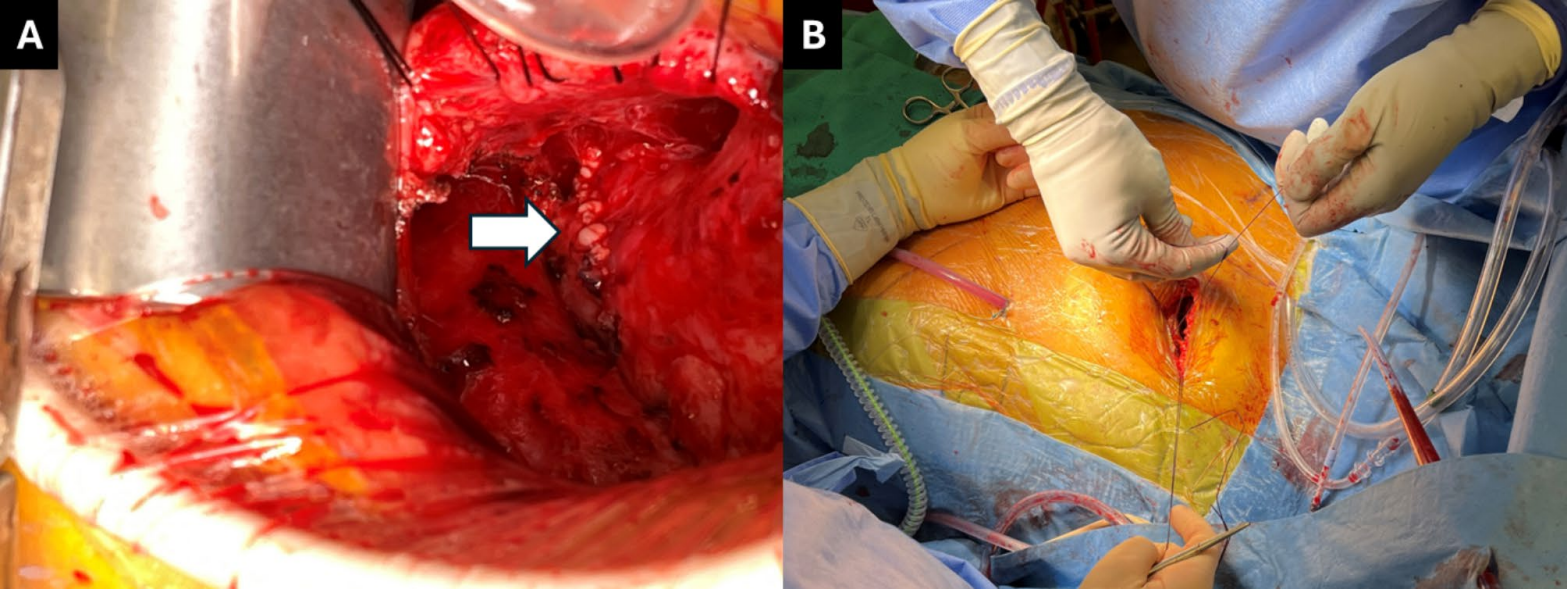
Operative images. (**A**) repaired pulmonary artery after excision of pulmonary artery mass (white arrow); (**B**) closure of left anterior mini-thoracotomy incision

Preoperative transesophageal echocardiogram demonstrated the mass without involvement of the pulmonic valve nor with any associated valvular dysfunction (Figure 1C). A 4cm left anterior mini thoracotomy was performed in the second intercostal space. Using lung isolation, extensive adhesiolysis was performed to mobilize the lung and chest wall off the pulmonary artery. Following adequate mobilization, the patient was placed on cardiopulmonary bypass using the femoral artery and vein via Seldinger technique utilizing percutaneous pre-closure devices. A longitudinal incision was made over the prior pulmonary artery anastomosis, careful to avoid the pulmonic valve. The excised mass was found to be growing from the posterior suture line and sent for frozen specimen evaluation. There was a noted communication between the posterior wall of the pulmonary artery and the left atrial appendage, unroofed by excision of the mass, which was oversewn. After confirming the sample was without malignancy, the pulmonary artery was closed, and the patient weaned from cardiopulmonary bypass (Figure 2). The patient was extubated on postoperative day 1 and was discharged postoperative day 4.

**Fig. 3:**
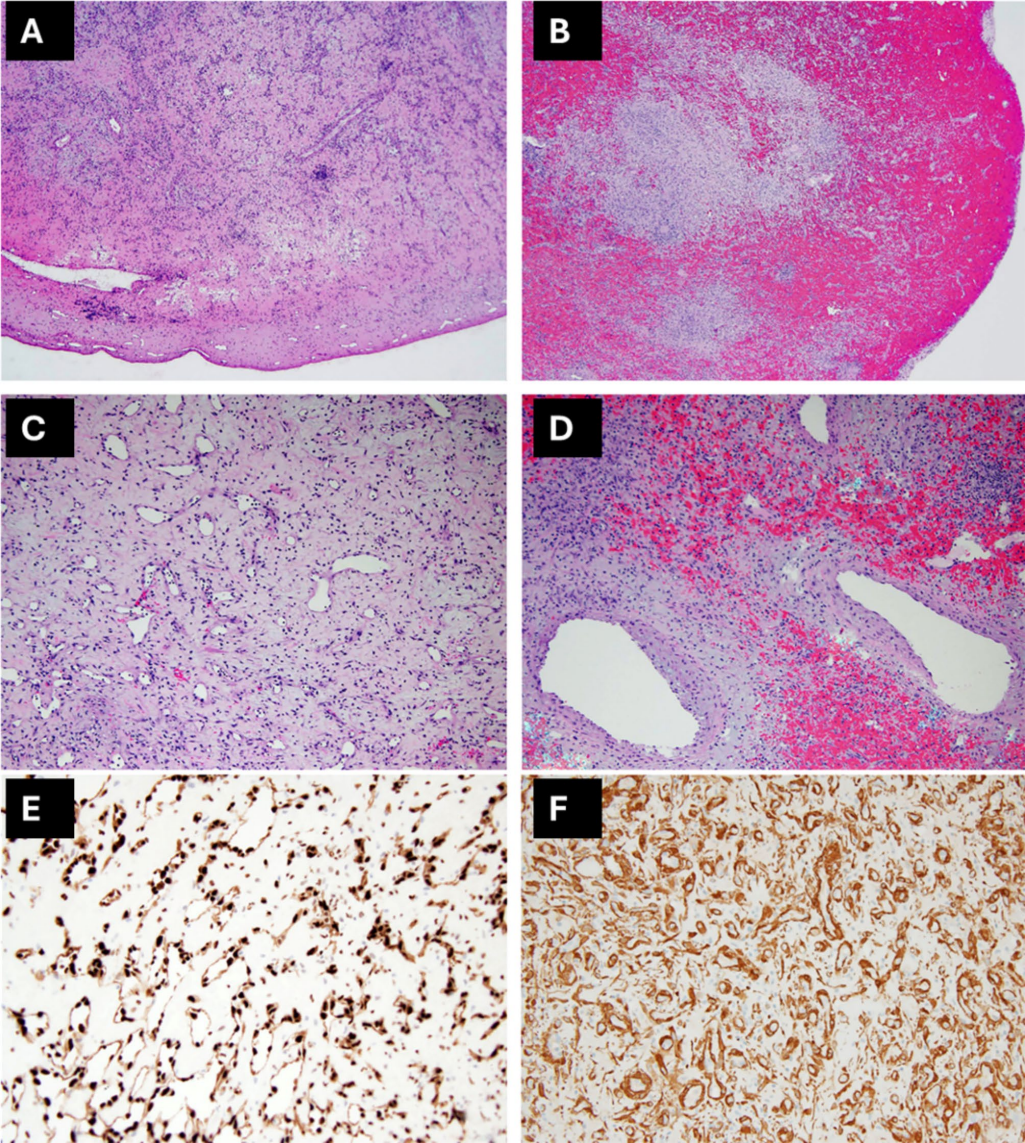
Histologic and immunochemical staining images of pulmonary artery mass. (**A**) well circumscribed lesion composed of numerous small and medium sized blood vessels; (**B**) areas of hemorrhage; (**C**) disorganized proliferation of thin-walled vascular channels; (**D**) proliferation of larger venous channels; (**E**) stain for endothelial transcription factor related gene highlighting endothelial cells in vascular proliferation; **D**) smooth muscle actin staining pericytes along walls of capillaries

Histopathologically, the lesion was well circumscribed and composed of clusters of thin-walled channels, numerous small vessels (arteries, veins, and indeterminate channels), larger irregular venous channels, and inflammatory infiltrate with lymphoplasmacytic aggregates. Cords and nests of endothelial cells with moderate amount of eosinophilic cytoplasm and round nuclei with inconspicuous nucleoli set in a fibromyxoid stroma were also noted (Fig. 3A-D). Immunohistochemical stains for endothelial transcription factor related gene highlighted the endothelial cells in vascular proliferation (Fig. 3E). Pericytes along the walls of capillaries were strongly positive for smooth muscle actin (Fig. 3F). Calretinin stain was negative, ruling out cardiac myxoma. Solid tumor next-generation sequencing (NGS) panel was positive for *PIK3CA* p.(E545K) mutation. Overall, these pathologic features were most consistent with a *PIK3CA* mutated vascular overgrowth. FusionPlex next-generation sequencing panel was negative for gene rearrangements/fusions seen in vascular neoplasms such as pseudomyogenic hemangioendothelioma (*FOSB* rearrangement) and epithelioid hemangioendothelioma (*WWTR1-CAMTA1* and *YAP1-TFE3* fusions).

## Comment

Malignancies following heart transplantation have been well described [1]. Fortunately, despite the patient’s oncologic history and concerning findings on diagnostic imaging, the excised mass was benign. There are currently no guidelines for malignancy screening surveillance, regardless of pre-transplant oncologic history. However, due to the increased malignancy risk, institutions like ours perform history specific oncologic surveillance; for example, this patient continues to have annual screening right-sided mammograms. However, this vascular tumor would not have been identified on any oncologic surveillance studies.

Mutations in *PIK3CA E545K* are frequent oncogenes, particularly in breast cancer [3]. While this patient did not undergo comprehensive testing for her breast cancer, it’s possible these oncogenes were involved with her oncologic past. Moreover, patients with *PIK3CA* were found to have increased risk of thrombosis, possibly due to activation of vascular endothelial cellular adhesion molecules [4]. In retrospect, while it could be associated with her cancer, she did have a history of thromboembolic disease with lower and upper extremity deep vein thromboses and bilateral pulmonary embolus that may have been associated with the mutation. Given the finding of a small defect in the pulmonary artery that may have been a fistulous formation with the left atrial appendage, it’s possible that the vascular overgrowth was triggered by endothelial injury, and she may be at risk for developing further masses in areas of endothelial injury.

While the mass was concerning for malignancy, there were no hemodynamic abnormalities or valvular dysfunction associated with the mass. Given the risks and increased mortality associated with reoperative cardiac surgery [5], we held a multi-disciplinary discussion on the benefits of performing reoperative cardiac surgery for diagnostic purposes. Ultimately, we decided it was worth pursuing an operation for an excisional biopsy given the absence of other targets for biopsy. Left anterior mini thoracotomy has been shown to be a safe approach for treating pulmonic valve disease [6]. Therefore, we decided to spare the patient a redo sternotomy and attempted the much less morbid, alternative approach. The patient had an uneventful recovery and was discharged 4 days after surgery.

The minimally invasive approach allowed us to remove the mass, find a diagnosis, and subsequently alter her management with minimal morbidity. While the *PIK3CA* mutation was not elucidated during her breast cancer evaluation earlier in life, now that it has been identified, she can be screened and treated for thromboembolic sequelae more aggressively moving forward. Given the findings, we decided to discharge her on oral anticoagulation.

## Data Availability

No datasets were generated or analysed during the current study.
